# Does kidney transplantation with a standard or expanded criteria donor improve patient survival? Results from a Belgian cohort

**DOI:** 10.1093/ndt/gfab024

**Published:** 2021-02-26

**Authors:** Rachel Hellemans, Anneke Kramer, Johan De Meester, Frederic Collart, Dirk Kuypers, Michel Jadoul, Steven Van Laecke, Alain Le Moine, Jean-Marie Krzesinski, Karl Martin Wissing, Kim Luyckx, Marieke van Meel, Erwin de Vries, Ineke Tieken, Serge Vogelaar, Undine Samuel, Daniel Abramowicz, Vianda S Stel, Kitty J Jager

**Affiliations:** 1 Department of Nephrology, Antwerp University Hospital, Edegem, Belgium; 2 Department of Medical Informatics, ERA-EDTA Registry, Amsterdam UMC, Academic Medical Center, Amsterdam Public Health Research Institute, Amsterdam, The Netherlands; 3 Nederlandstalige Belgische Vereniging voor Nefrologie, Sint-Niklaas, Belgium; 4 Groupement des Néphrologues Francophones de Belgique, Liège, Belgium; 5 Department of Nephrology, University Hospitals Leuven, Leuven, Belgium; 6 Département de Néphrologie, Cliniques Universitaires Saint-Luc, Bruxelles, Belgium; 7 Department of Internal Medicine, Renal Division, Ghent University Hospital, Ghent, Belgium; 8 Département de Néphrologie, Hôpital Erasme–Université Libre de Bruxelles, Bruxelles, Belgium; 9 Centre Hospitalier Universitaire de Liège, Service de Néphrologie, Liège, Belgium; 10 Department of Nephrology, Universitair Ziekenhuis Brussel, Brussel, Belgium; 11 Department of Informatics, Antwerp University Hospital, Edegem, Belgium; 12 Eurotransplant International Foundation, Leiden, The Netherlands; 13 Eurotransplant International Foundation, Leiden, The Netherlands

**Keywords:** dialysis, elderly, expanded criteria donor, kidney transplantation, survival

## Abstract

**Background:**

Changes in recipient and donor factors have reopened the question of survival benefits of kidney transplantation versus dialysis.

**Methods:**

We analysed survival among 3808 adult Belgian patients waitlisted for a first deceased donor kidney transplant from 2000 to 2012. The primary outcome was mortality during the median waiting time plus 3 years of follow-up after transplantation or with continued dialysis. Outcomes were analysed separately for standard criteria donor (SCD) and expanded criteria donor (ECD) kidney transplants. We adjusted survival analyses for recipient age (20–44, 45–64 and ≥65 years), sex and diabetes as the primary renal disease.

**Results:**

Among patients ≥65 years of age, only SCD transplantation provided a significant survival benefit compared with dialysis, with a mortality of 16.3% [95% confidence interval (CI) 13.2–19.9] with SCD transplantation, 20.5% (95% CI 16.1–24.6) with ECD transplantation and 24.6% (95% CI 19.4–29.5) with continued dialysis. Relative mortality risk was increased in the first months after transplantation compared with dialysis, with equivalent risk levels reached earlier with SCD than ECD transplantation in all age groups.

**Conclusions:**

The results of this study suggest that older patients might gain a survival benefit with SCD transplantation versus dialysis, but any survival benefit with ECD transplantation versus dialysis may be small.

## Graphical Abstract

**Figure:**
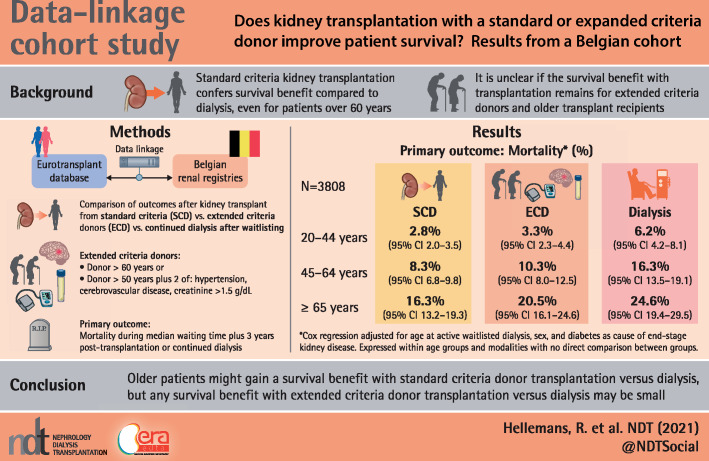

**KEY LEARNING POINTS**

**What was already known about this subject?**

- Older studies have shown a survival benefit with kidney transplantation compared with dialysis, even for patients >60 years of age. However, due to important evolutions such as older recipient age and the use of less-than-optimal-quality donors, it is unclear if the survival benefit with transplantation still holds true today.

**What this study adds?**

- Among patients ≥65 years of age waitlisted for a first deceased donor kidney transplant in Belgium, only standard criteria donor (SCD) transplantation provided a significant survival benefit compared with continued dialysis.
- The relative risk of mortality in the first months after transplantation was higher with expanded criteria donor (ECD) than with SCD transplantation.
- The results of this study suggest that older patients might gain a survival benefit with SCD transplantation versus dialysis, but any survival benefit with ECD transplantation versus dialysis may be small.

**What impact this may have on practice or policy?**

- Older transplant candidates should be informed that any survival benefit with ECD transplantation may be small.
- The acceptance of an ECD transplant should be carefully balanced against the risks of continued dialysis while waiting for a better donor offer.
- Larger European studies are needed to provide more precise estimates of the survival benefit with transplantation.

## INTRODUCTION

Since 1999, when Wolfe *et al.* [1] reported a survival benefit for US patients receiving a first deceased donor kidney transplantation as compared with waitlisted dialysis patients, kidney transplantation has been assumed to offer better survival. The results of that study motivated the choice of kidney transplantation for end-stage renal disease (ESRD), even for older patients. However, in that study only 15% (*n* = 6925) of the total study population was ≥60 years of age at the time of waitlisting, suggesting the possibility of a selection bias toward fit older patients being considered eligible for transplantation.

In the ensuing two decades, the kidney transplantation landscape has evolved profoundly. First, the median age of recipients of a deceased donor kidney increased from 45 years in 1990 to 56 years in 2016, according to Eurotransplant, an organ allocation organization that allocates deceased donor organs in Austria, Belgium, Croatia, Germany, Hungary, Luxembourg, The Netherlands and Slovenia [2]. Furthermore, in keeping with this higher median age, current transplant recipients are more likely to have more pretransplant comorbidities [3]. Because of organ shortages, older-donor kidneys and donor kidneys of suboptimal quality are being transplanted. In Eurotransplant, the median age of deceased kidney donors has risen steeply from 36 years in 1990 to 54 years in 2016 [2], and about one-third of them can now be classified as expanded criteria donors (ECDs) [2, 4]. By definition, ECD kidneys show inferior graft survival compared with those from standard criteria donors (SCDs), with a relative risk of graft failure of >1.7 [4]. Moreover, transplantation with an ECD compared with an SCD kidney negatively affects patient survival [5]. In parallel with these important changes in the transplantation field, improved management of chronic kidney disease and dialysis care has led to improved survival on dialysis in Europe and the USA [6, 7].

The result of these developments is a situation of more high-risk transplantations (older candidates, more comorbidities) with more donor kidneys of suboptimal quality even as survival on dialysis is improving. Recent studies that update outcomes with transplantation versus dialysis are quite limited, especially for Europe, and show diverging results [8–11]. Development of such studies is hampered because many countries lack a comprehensive registry that captures waitlist, transplantation and outcome data such as patient and graft survival.

In this study we analysed patient survival data for adults (age ≥20 years) waitlisted for a first deceased donor kidney transplantation in Belgium, a European country of 11 million inhabitants. We divided the population into three age groups and compared survival with either SCD or ECD transplantation versus continued dialysis. For this purpose we linked waitlist and donor data from Eurotransplant with patient survival data for Belgian ESRD patients from the two regional Belgian renal registries [Nederlandstalige Belgische Vereniging voor Nefrologie (NBVN) and Groupement des Néphrologues Francophones de Belgique (GNFB)] from the Dutch-speaking and French-speaking part of Belgium, respectively.

## MATERIALS AND METHODS

### Study population

**FIGURE 1:**
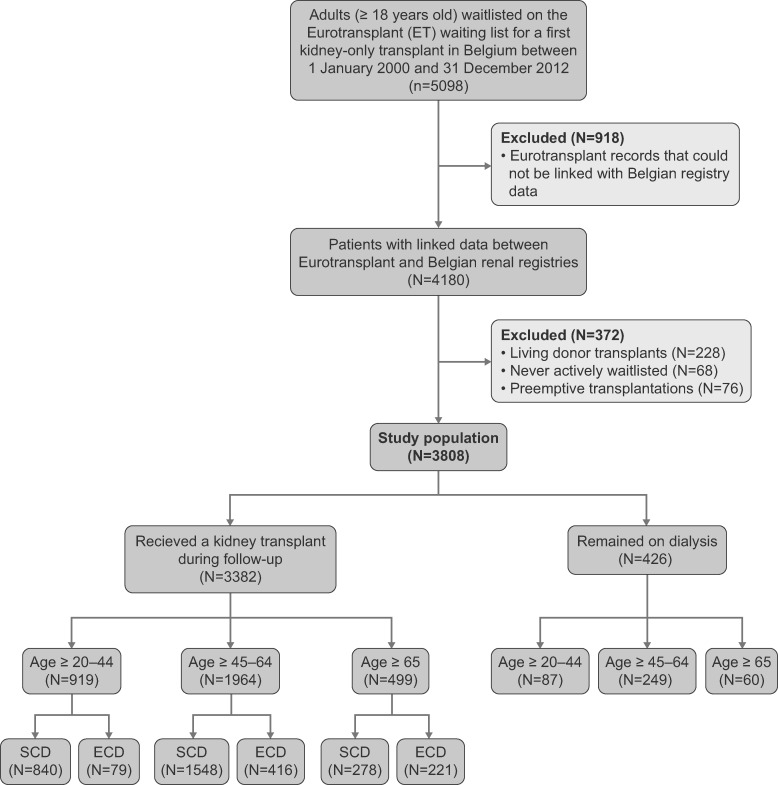
Study flowchart.

The study population was selected from 5098 patients ≥20 years of age who were registered on the Eurotransplant waitlist for a first kidney-only transplantation in Belgium between 1 January 2000 and 31 December 2012. The Eurotransplant database contains complete data sets about waitlisting and detailed donor data (mandatory data set), but survival data on transplant recipients are incomplete (not mandatory). Therefore we needed to merge the selected study population from the Eurotransplant database with data from two Belgium renal registries (NBVN and GNFB) containing follow-up data of patients on renal replacement therapy (e.g. mortality). With the explicit consent of NBVN, GNFB and all Belgian transplant centers for the use of these data, merging of the databases was performed at Eurotransplant and only strictly anonymized data were made available for the analysis. Starting from the selected Eurotransplant dataset containing 5098 patients, we were able to find the corresponding patient in the Belgian renal registry database in 4180 (82%) of the cases (Figure 1). Merging was done according to a specifically designed algorithm. To explore the reasons why some Eurotransplant records could not be linked to data from the Belgian registries, we previously performed a feasibility study based on the Antwerp cohort (representing ∼10% of the waitlisted population in Belgium); failed linking was due to data entry differences (e.g. spelling errors) between databases, patients who were waitlisted in Belgium but received dialysis abroad, patients on the waitlist but not yet on dialysis during the study period, patients ≥20 years of age at waitlisting who were still treated by paediatric nephrologists (who in Belgium do not register patients in the national renal registries) or patients not captured in the national Belgian renal registries because of errors (unpublished data). Of the original 4180 patients, 372 were excluded: 228 patients received a living donor transplantation, 68 patients never received active waitlist status after registration at Eurotransplant and 76 had pre-emptive transplantations. Therefore the final dataset for analysis contained 3808 patients.

### Patient groups and definitions

Patients were followed from the moment they were both on dialysis and actively waitlisted for a first deceased kidney donor transplant. Patients who were transplanted during the study period are referred to as ‘transplant recipients’, whereas those who were not transplanted are referred to as ‘patients remaining on dialysis’. Patients were divided into age categories (≥20–44, ≥45–64 and ≥65 years) according to their age at the time of active waitlisting while on dialysis. ECDs were defined as all donors >60 years of age or donors >50 years meeting at least any two of the following criteria: hypertension, cerebrovascular cause of death or serum creatinine level >1.5 mg/dL before organ recovery [4]. SCDs were donors who did not meet the ECD criteria.

### Outcomes

The main outcome was expressed as mortality probabilities. For patients who received a kidney transplant, the mortality on dialysis during the median waiting time for a kidney transplant plus the mortality 3 years after transplantation was taken as the primary outcome. For patients remaining on dialysis, the primary outcome was mortality during the median waiting time plus mortality during the 3 years thereafter. Median waiting time was defined as the time at which 50% of the waiting list population was transplanted. Median waiting time plus 3 years was chosen as the most appropriate length of follow-up because of the small number of patients (<10%) remaining in the dialysis subgroups after this period. For transplanted patients, outcomes were also analysed separately for those receiving an ECD or SCD kidney transplant.

### Statistical analysis

Data on baseline characteristics are presented as medians (25th–75th percentiles) or as percentages, unless otherwise specified. To compare the baseline characteristics of the different study groups, we used chi-squared or non-parametric tests, where applicable.

Simply beginning from waitlisting and then comparing mortality probabilities between those who are eventually transplanted and those remaining on dialysis would result in immortal time bias during the waiting time for the transplanted group, as these patients will then, by definition, survive until the day they receive a kidney transplantation. Therefore, calculation of mortality probabilities for patients remaining on dialysis or receiving a kidney transplant consisted of several steps. First, we used the cumulative incidence competing risk method to calculate the median waiting time for each of the age categories, from the start of being actively waitlisted and on dialysis until transplantation, with death or permanent delisting as competing events. Second, we calculated mortality rates during the median waiting time for each of the age categories using Cox regression. For these analyses, the survival time started with the date of active waitlisted dialysis and ended with the date of death or a censored observation (delisting because of transplantation, recovery or loss to follow-up), the end of follow-up (median waiting time per age category) or the end of the study period (31 December 2015). In these survival analyses, we did not censor for delisting due to poor health status, because it is expected that several patients who are delisted due to poor health may die shortly thereafter, possibly in the study period. Therefore, censoring for delisting due to poor health would have resulted in an overestimation of survival. Models were adjusted for age at active waitlisted dialysis, sex and diabetes as the cause of ESRD. Analyses included the total group of patients and we obtained age stratum–specific mortality rates by applying fixed values for age (median age by each age category, i.e. 35.2 years for age category ≥20–44, 55.2 years for age category ≥45–64 and 68.5 years for age category ≥65), sex (60%) and diabetes (2%) to the Cox regression models. Third, for patients remaining on dialysis, the adjusted mortality risk during the median waiting time was added to the adjusted mortality risk on dialysis 3 years thereafter. This was calculated using the same methods as described above, except that survival time was extended to 3 years after the median waiting time. This means that the analysis is based on the entire population with censoring at the time of transplantation. Again, patients were not censored at the time of delisting from the waiting list due to poor health status. For patients who received a kidney transplant during follow-up, the adjusted mortality risk during the median waiting time was added to the adjusted mortality from transplantation until 3 years thereafter. For the calculation of the adjusted mortality from transplantation until 3 years thereafter, survival time started with the date of transplantation and ended with the date of death, a censored observation (loss to follow-up), end of follow-up (3 years after transplantation) or the end of the study period (31 December 2015). Cox regression analysis was used, again with adjustment for age at active waitlisted dialysis, sex and diabetes as the cause of ESRD and using the fixed values for age, sex and diabetes as described above. To calculate the adjusted relative mortality risk after transplantation versus the mortality of those remaining on dialysis over time, we divided the monthly cumulative mortality from transplantation by the cumulative mortality of those still on dialysis from the median waiting time. Mortality rates were adjusted for fixed values of age, sex and diabetes. All statistical analyses were performed using SAS software version 9.4 (SAS Institute, Cary, NC, USA).

## RESULTS

### Population characteristics

The final dataset for analysis contained 3808 patients. Overall, 3341 of these patients initiated chronic dialysis treatment before active waitlisting and 467 patients did so on the day of or after active waitlisting. Patients were followed until 31 December 2015. The median follow-up time was 8.5 years (25th–75th percentiles 5.5–12.4) in the 20–44-year age group, 7.4 years (25th–75th percentiles 4.5–10.5) in the 45–64-year age group and 5.5 (25th–75th percentiles 3.5–8.5) in the ≥65-year age group.

Of a total of 3808 waitlisted patients included in the study, 3382 (88.8%) underwent a kidney transplant during follow-up. In the youngest age group of transplant recipients (20–44 years; *n* = 919), 91% received an SCD kidney. In the 45–64-year age group (*n* = 1964), 79% received an SCD kidney. In the oldest age group (≥65 years; *n* = 499), 56% received an SCD kidney (Figure 1).

Table 1 summarizes the baseline characteristics of the study population. Transplant recipients were significantly younger and more likely to have autosomal dominant polycystic kidney disease or glomerulonephritis as causes of ESRD, whereas those remaining on dialysis were older and more likely to have ESRD caused by diabetes or hypertensive/vascular disease. Recipients of ECD kidneys were older than SCD kidney recipients, more likely to have spent more time on dialysis before active waitlisting and more likely to have ESRD caused by hypertension/vascular disease. ECD recipients were less likely to have ESRD because of glomerulonephritis or to have been waitlisted before starting dialysis. Donor characteristics are summarized in Supplementary data, File S1.

**Table 1.** Baseline characteristics

	Transplant recipients					
Patient characteristics	Total TX	SCD	ECD	P-value**	Waitlisted patients remaining on dialysis (*n* = 426)	P-value*
	(*n* = 3382)	(*n* = 2666)	(*n* = 716)			
Age^a^ (years), median (25–75th percentile)	53 (44–61)	51 (42–59)	61 (52–66)	<0.001	56 (47–63)	<0.001
Age distribution^a^, *n* (%)						
≥20–44	919 (27)	840 (32)	79 (11)	<0.001	87 (20)	0.003
≥45–64	1964 (58)	1548 (58)	416 (58)	0.986	249 (58)	0.881
≥65	499 (15)	278 (10)	221 (31)	<0.001	90 (21)	<0.001
Male, *n* (%)	2137 (63)	1694 (64)	443 (62)	0.411	256 (60)	0.213
Cause of renal failure, *n* (%)						
ADPKD	626 (18)	494 (18)	132 (19)	0.954	57 (13)	0.009
Diabetes	60 (2)	48 (2)	12 (2)	0.823	25 (6)	<0.001
Hypertension/vascular disease	315 (9)	227 (9)	88 (12)	0.002	39 (9)	0.915
Glomerulonephritis	969 (29)	782 (29)	187 (26)	0.091	92 (22)	0.002
Uncertain aetiology	440 (13)	347 (13)	93 (13)	0.985	48 (11)	0.311
Other	972 (29)	768 (29)	204 (28)	0.868	165 (39)	<0.001
Dialysis modality^b^, *n* (%)						
Haemodialysis	2151 (64)	1680 (63)	471 (66)	0.172	279 (65)	0.444
Peritoneal dialysis	669 (20)	525 (20)	144 (20)	0.802	76 (18)	0.341
No dialysis	404 (12)	334 (13)	70 (10)	0.044	64 (15)	0.068
Missing data	158 (4)	127 (4)	31 (4)	0.625	7 (2)	0.004
Days on dialysis before active waitlisting, median (25–75th percentile)	253 (121–510)	246 (117–505)	282 (142–526)	0.023	291 (137–530)	0.099

The total study population consists of 3808 patients. Baseline characteristics are shown separately for patients remaining on dialysis during follow-up and those who were transplanted during follow-up.

*P-value comparing patients remaining on dialysis versus transplanted patients. **P-valuecomparing SCD and ECD transplantation.

a Age at the moment when the patient was both actively waitlisted and on dialysis (whichever came second).

b At the time of registration on the waiting list.

ADPKD, autosomal dominant polycystic kidney disease; TX, transplantation.

### Time on the active waiting list and mortality during waiting time

The median waiting time on the active waiting list for kidney transplantation while on dialysis was significantly longer in the younger age categories. In the youngest age group, the median waiting time was 22.4 months (25th–75th percentiles 8.9–42.0); in the middle age group, the median waiting time was 18 months (25th–75th percentiles 6.9–38.8) and in the oldest age group, the median waiting time was 11.7 months (25th–75th percentiles 3.6–28.7) (P < 0.0001 among all groups).

In the youngest age group, mortality during the median waiting time (22 months) was 1.1% [95% confidence interval (CI) 0.7–1.5]; in the middle age group, mortality during the median waiting time (18 months) was 2.3% (95% CI 1.7–2.9) and in the oldest age group, mortality during the median waiting time (12 months) was 2.5% (95% CI 1.6–3.3). There were only minor differences in median waiting time between those who eventually received an SCD versus ECD transplant within each age group, with no substantial impact on mortality during the median waiting time (Supplementary data File S2,).

### Mortality during the median waiting time plus 3 years post-transplantation or continued dialysis

**FIGURE 2:**
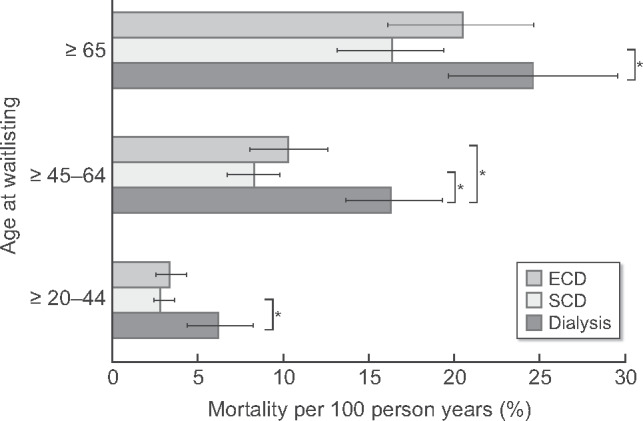
Mortality during median waiting time (median waiting time according to age group: age ≥20–44 years: 22 months; age ≥45–64 years: 18 months; age ≥65 years: 12 months) plus 3 years post-transplantation or continued dialysis. Cox regression analysis adjusted for age, sex, diabetes as the primary kidney disease (for more details: see Methods section). *No overlap between 95% CIs.

In the youngest age group, when compared with those remaining on dialysis, mortality was statistically significantly lower for those transplanted with an SCD (Table 2 and Figure 2) and there was a clear trend towards a lower mortality for those transplanted with an ECD.

**Table 2.** Mortality during the median waiting time^a^ plus 3  years post-transplantation or continued dialysis

	Mortality, % (95% CI)
Age ≥22–44 years	
ECD	3.3 (2.3–4.4)
SCD	2.8 (2.0–3.5)
Dialysis	6.2 (4.2–8.1)
Age ≥45–64 years	
ECD	10.3 (8.0–12.5)
SCD	8.3 (6.8–9.8)
Dialysis	16.3 (13.5–19.1)
Age ≥65 years	
ECD	20.5 (16.1–24.6)
SCD	16.3 (13.2–19.3)
Dialysis	24.6 (19.4–29.5)

Cox regression analysis adjusted for age, sex, primary renal disease.

a Median waiting time according to age group: age 22–44 years: 22 months; age 45–64 years: 18 months; age ≥65 years: 12 months.

In the middle age group, those transplanted with an ECD or SCD had a statistically significantly lower mortality than those remaining on dialysis. In addition, there was a clear trend towards a lower mortality in those transplanted with an SCD compared with those who received an ECD transplant.

In the oldest age group, mortality was statistically significantly lower for those transplanted with an SCD than for those remaining on dialysis. For those transplanted with an ECD, the mortality tended to be lower than for patients remaining on dialysis, while it tended to be higher compared with patients who received an SCD transplant.

**FIGURE 3:**
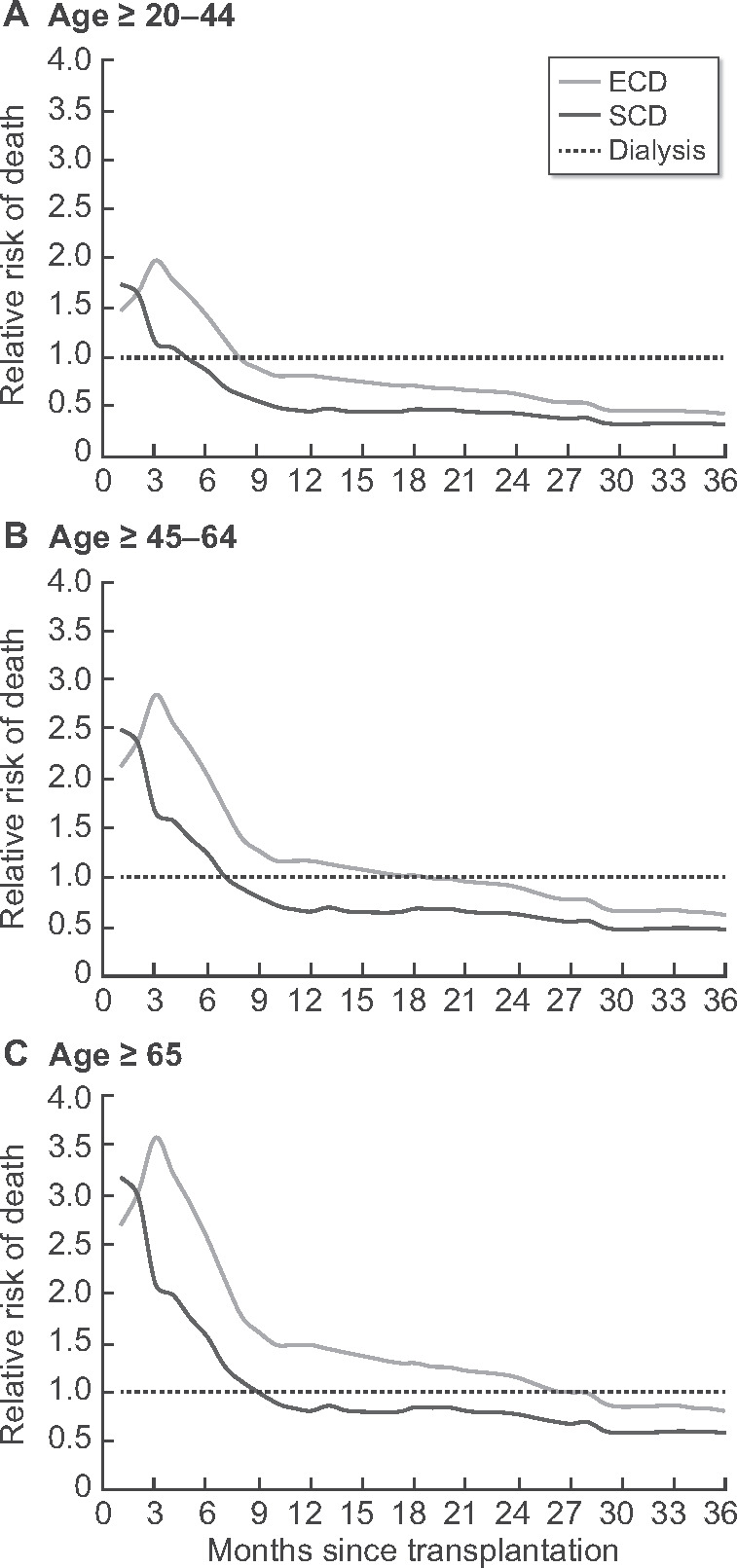
Adjusted relative risk of death over time after SCD or ECD donor transplantation compared with patients remaining on dialysis by age category. Adjusted relative mortality risk among 3382 recipients of a first deceased donor transplant compared with the reference group of 1852 patients still on dialysis after the median waiting time (median waiting time according to age group: age ≥20–44 years: 22 months; age ≥45–64 years: 18 months; age ≥65 years: 12 months). Mortality rates are adjusted using fixed values for age, sex and diabetes as cause of ESRD. Outcomes are shown separately for recipients of an SCD and ECD. Dx, patients remaining on dialysis.

Finally, we compared the relative mortality risk over time of SCD and ECD transplantation with the mortality risk in patients remaining on dialysis (Figure 3). In all age groups, transplantation was associated with an increased risk of death in the first months after transplantation but with a lower risk of death later in the follow-up period. The mortality risk was consistently higher with ECD than with SCD transplantation in all age groups, with a peak at 3 months post-transplant. Correspondingly, the period of increased mortality risk in transplanted patients versus patients remaining on dialysis was consistently longer for recipients of ECD kidneys than for SCD kidney recipients: it was 7 versus 4 months for patients ages 20–44 years, 18 versus 7 months for those ages 45–60 years and 26 versus 8 months in the ≥65-year age group.

## DISCUSSION

We analysed long-term survival outcomes after kidney transplantation versus dialysis in a cohort of Belgian patients on the Eurotransplant waitlist. Our findings suggest that even patients >65 years of age experience a survival benefit with transplantation, at least when transplanted with an SCD kidney. Outcomes are less favourable when older patients are transplanted with an ECD kidney. In fact, we did not observe a statistically significant difference in survival between older patients receiving an ECD transplantation and those remaining on dialysis, despite a trend favouring ECD transplantation.

Our study showed that ECD transplantation in Belgium was associated with a higher early post-transplant mortality risk compared with SCD transplantation, confirming recent US findings [12]. This increased risk of early post-transplant mortality with ECD versus SCD transplantation may result from an increased risk for delayed or poor early graft function, which in turn can lead to prolonged hospitalization. This situation gives rise to difficulties with fluid and immunosuppressive drug management and with a higher risk for complications, such as infections and cardiovascular events, and for acute rejection [13–15].

Do these findings together mean that we should avoid transplanting older patients with ECD? This issue is complex. First, the dichotomous classification of SCD versus ECD very much oversimplifies the highly heterogeneous pool of donors. Some ECD kidneys may lead to acceptable outcomes, while others will do poorly [16]. A more granular score of donor quality, such as the Kidney Donor Risk Index developed in the USA, may be more appropriate [17]. More research is needed to assess the added value of this index in estimating transplant outcomes in Europe. Pre-implantation biopsies, as performed by some transplant centres, could be another way to better assess the quality of older-donor kidneys [18]. Second, although it may be tempting to keep the patient on the waiting list until there is an SCD donor offer, patients tend to accumulate dialysis-related complications, which could lead to withdrawal from the waitlist and a lost chance of ever getting transplanted. In addition, even when they ultimately do receive an SCD transplant, a longer dialysis period will remain a burden that may negatively affect post-transplant survival [19, 20]. Finally, even in the absence of a survival benefit, ECD transplantation could lead to better physical, mental and social well-being compared with dialysis, which may be the most important motivation for choosing transplantation for older dialysis patients.

Other studies comparing outcomes with transplantation versus dialysis are limited and show somewhat contradictory results. An analysis from a US population from the early 2000s suggested a survival benefit with transplantation, even for recipients >70 years of age and with ECDs [11]; however, the risks vary with recipient comorbidities and donor type [12]. These findings cannot be generalized to Europe, where survival outcomes on dialysis and after transplantation appear to be better than in the USA [21, 22]. Results of two European studies, from France and Catalonia, also suggested a survival benefit with transplantation, even in older recipients and even with the use of ECDs [8, 9]. On the other hand, a recent analysis from the Netherlands showed no survival benefit in patients >65 years of age transplanted with kidneys from older donors [10].

It is difficult to interpret the diverging results of the studies, not only because of the large heterogeneity between the populations, but also because of the varying methodology. It is obviously not possible to conduct a randomized trial to assess the survival benefit of transplantation. The most appropriate approach is to compare outcomes of transplanted patients with those of a control group of also waitlisted dialysis patients who are not (yet) transplanted; these patient groups are most likely to share common features. However, some selection bias will remain because healthier patients are somewhat more likely to be transplanted than those who are less fit, because the latter tend to have more complications and spend more time being temporarily considered ‘non-transplantable’. To minimize this bias, we adjusted for age, sex and diabetes as the primary renal disease, but we lacked the data to adjust for comorbidities or for time intervals spent being ‘non-transplantable’.

Selection of the appropriate starting point for analysing the effect of transplantation on patient survival represents an additional methodological difficulty, often only vaguely addressed in other studies [23]. Using the time of transplantation as the starting point makes the starting point for those who are not transplanted unclear. Thus, setting the start of dialysis for this control group’s starting point would result in lead-time bias. Beginning the analysis at the time of waitlisting for both groups seems more appropriate. However, beginning from waitlisting and then comparing outcomes between those who are transplanted and those remaining on dialysis would result in immortal time bias for the transplanted group, as these patients will survive until the day of kidney transplantation anyway. For this reason, we decided to first calculate mortality during the median waiting time, with waiting time for both the transplanted and non-transplanted patients based on a competing risk analysis. We then added this result to the 3-year mortality risk after transplantation and compared that outcome to an equal amount of time (median waiting time + 3 years) in the dialysis control group. Finally, we kept patients in the survival analysis if they were delisted because of poor health status, because censoring patients at delisting would have yielded an overestimation of survival on dialysis.

We acknowledge that our methodology has several remaining limitations. First, to maximize the power of our analyses, we performed survival analyses on the total group rather than separate analyses per age group. Then, age stratum–specific mortality rates were obtained by applying age stratum–specific fixed values for age, sex and diabetes mellitus in the Cox regression models. However, this approach uses the regression coefficients for age, sex and diabetes mellitus that were based on the entire sample and may therefore not entirely apply within the different patient subgroups. Also, mortality may depend on other factors that we have not adjusted for. Second, we chose to use mortality during the median waiting time as an approximation to overcome immortal time bias in the transplanted group. However, in reality, waiting time and the corresponding mortality during that waiting time differs between individuals. Finally, in the analysis of survival, dialysis patients undergoing dialysis who were censored at kidney transplantation may be healthier and may therefore have better survival prospects compared with those staying on dialysis, which may have resulted in an overestimation of mortality on dialysis.

Other limitations of this study include the possibility of incomplete registry data. It might be easier to keep track of patients in a dialysis unit than those who are transplanted and switch centres more easily, thus being more likely to be lost to follow-up. Incomplete data carry the risk of underreporting death with a functional graft, which also could have led to an overestimation of the survival benefit of transplantation. The sample size was relatively small, especially in the oldest age group. Also, the dialysis control group diminished quickly as a result of the high transplantation rate. These factors yielded survival estimates with relatively wide CIs and prevented a separate analysis according to transplantation from either a donor after brain death (DBD) or a donor after circulatory death (DCD). It is not clear whether such a further differentiation would have altered the conclusions. A recent analysis from the Netherlands showed no difference in 5-year patient survival between those receiving a DBD or a DCD transplant, even in patients ≥65 years of age receiving a kidney from a donor ≥65 years of age [10]. Nevertheless, reports on differential long-term outcomes with either DBD or DCD transplantation are conflicting [24–26] and we believe stratifying the analysis according to DBD or DCD donation would be desirable in a larger study. Finally, the relatively short waiting times in Belgium may have an impact on the prognosis after transplantation [19, 20] and therefore the conclusions from our study may not be generalizable to countries with much longer waiting times. Also, as the survival benefit with transplantation continuously evolves due to changes in dialysis and transplantation care, our study findings may not be valid beyond the time period studied.

An important strength of this study is that it showed for the first time in Belgium, the feasibility of linking national renal registry data with that of a large transplant registry, such as Eurotransplant. This achievement opens the possibility of broadening the combined national registry/Eurotransplant dataset to include other European countries and establishing long-term scientific epidemiological collaborations involving Eurotransplant and other transplant organizations to investigate kidney transplantation in Europe. Furthermore, we aimed for a transparent and detailed methodology description that provides the opportunity for researchers to repeat the analyses in other cohorts and directly compare outcomes among cohorts.

In conclusion, the results of this study suggest that older patients might gain a survival benefit with SCD transplantation versus dialysis, but any survival benefit with ECD transplantation versus dialysis may be small. These findings should be interpreted with caution and need to be re-evaluated and studied in more depth in a larger European cohort to provide more precise estimates of the survival benefit with transplantation and to enable a better prediction at the level of the individual transplant candidate and his or her potential donor.

## SUPPLEMENTARY DATA

Supplementary data are available at ndt online.

## Supplementary Material

gfab024_Supplementary_Data
