# Supplementary material for: Does kidney transplantation with a standard or expanded criteria donor improve patient survival? Results from a Belgian cohort
Source: Nephrol Dial Transplant. 2021 Feb 26;36(5):918–26. doi: 10.1093/ndt/gfab024 (PMC8075371; doi:10.1093/ndt/gfab024)
Supplement: gfab024_Supplementary_Data [file gfab024_supplementary_data.pdf]

Supplementary table 1: Donor characteristics

Data are presented as number of patients (percentage) or as median (25-75 percentile), unless indicated otherwise

Abbreviations:TX: transplantation, SCD: standard criteria donors, ECD: expanded criteria donors, CVA: cerebrovascular accident, DBD: donation after brain death , DCD: donation after circulatory death, KDRI: kidney donor risk index, CIT: cold ischemia time. P-value*: comparing SCD and ECD transplantation. [a]: KDRI calculated using the formula by Rao et al.based on the 10 donor-derived factors (Rao PS, Schaubel DE, Guidinger MK, et al. A comprehensive risk quantification score for deceased donor kidneys: the kidney donor risk index. *Transplantation*. 2009;88(2):231-236.)

	TOTAL TX N=3382	SCD N=2666	ECD N=716	p-value*
**Age(years)**	47 (36-56)	43 (31-51)	62 (60-66)	<0.0001
**Male sex**	1910 (56)	1576 (59)	334 (47)	<0.0001
**Hypertension history:**				
**Yes**	711 (21)	325 (12)	386 (54)	
**No**	2066 (61)	1819 (68)	247 (35)	<0.0001
**Unknown**	605 (18)	522 (20)	83 (11)	
**CVA as cause of death**	1129 (33)	686 (26)	443 (62)	<0.0001
**Serum creatinine:**				
**mg/dl**	0.79 (0.60-1.00)	0.79 (0.60-1.00)	0.78 (0.60-0.97)	0.09
**>1.5 mg/dl**	124 (3.7)	97 (3.6)	27 (3.8)	0.82
**Diabetes:**				
**Yes**	29 (1)	16 (1)	13 (2)	
**No**	2433 (64)	1891 (71)	542 (76)	0.002
**Unknown**	1346 (35)	759 (28)	161 (22)	
**Deceased donor type:**				
**DBD**		2270 (85)	637 (89)	0.009
**DCD**		396 (15)	79 (11)	
**KDRI[a]**		1.08 (0.92-1.27)	1.67 (1.56-1.92)	<0.0001
**CIT (hours), mean (SD)**		14.6 (4.8)	13.9 (4.9)	0.001
**HLA mismatches 0-6, mean (SD)**		2.3 (1.1)	2.8 (1.3)	<0.0001

Supplementary table 2: **Median waiting time for recipients of a SCD or ECD transplants, according to age category, and corresponding mortality during median waiting time.**

Age category	Donor type	Median waiting time (months)	Mortality during median waiting time, % (95 % CI)
**≥20 - 44**	SCD	20,2	99.1 (98.8-99.4)
	ECD	16,5	99.3 (99.1-99.6)
**≥45 - 64**	SCD	15,0	98.1 (97.5-98.6)
	ECD	15,9	97.9 (97.4-98.5)
**≥65**	SCD	7.1	98.6 (98.0 -99.2)
	ECD	10,9	97.8 (97.0-98.6)

Mortality was calculated by Cox regression analysis adjusted for age, sex, diabetes as primary renal disease (for more details: see methods section).
